# Multiclass classification of whole‐body scintigraphic images using a self‐defined convolutional neural network with attention modules

**DOI:** 10.1002/mp.15196

**Published:** 2021-09-14

**Authors:** Qiang Lin, Chuangui Cao, Tongtong Li, Yongchun Cao, Zhengxing Man, Haijun Wang

**Affiliations:** ^1^ School of Mathematics and Computer Science Northwest Minzu University Lanzhou China; ^2^ Key Laboratory of Streaming Data Computing Technologies and Application Northwest Minzu University Lanzhou China; ^3^ Key Laboratory of China's Ethnic Languages and Information Technology of Ministry of Education Lanzhou China; ^4^ Department of Nuclear Medicine Gansu Provincial Hospital Lanzhou China

**Keywords:** attention mechanism, bone scintigraphy, convolutional neural network, medical image analysis, multiclass classification

## Abstract

**Purpose:**

A self‐defined convolutional neural network is developed to automatically classify whole‐body scintigraphic images of concern (i.e., the normal, metastasis, arthritis, and thyroid carcinoma), automatically detecting diseases with whole‐body bone scintigraphy.

**Methods:**

A set of parameter transformation operations are first used to augment the original dataset of whole‐body bone scintigraphic images. A hybrid attention mechanism including the spatial and channel attention module is then introduced to develop a deep classification network, Dscint, which consists of eight weight layers, one hybrid attention module, two normalization modules, two fully connected layers, and one softmax layer.

**Results:**

Experimental evaluations conducted on a set of whole‐body scintigraphic images show that the proposed deep classification network, Dscint, performs well for automated detection of diseases by classifying the images of concerns, achieving the accuracy, precision, recall, specificity, and *F*‐1 score of 0.9801, 0.9795, 0.9791, 0.9933, and 0.9792, respectively, on the test data in the augmented dataset. A comparative analysis of Dscint and several classical deep classification networks (i.e., AlexNet, ResNet, VGGNet, DenseNet, and Inception‐v4) reveals that our self‐defined network, Dscint, performs best on classifying whole‐body scintigraphic images on the same dataset.

**Conclusions:**

The self‐defined deep classification network, Dscint, can be utilized to automatically determine whether a whole‐body scintigraphic image is either normal or contains diseases of concern. Specifically, better performance of Dscint is obtained on images with lesions that are present in relatively fixed locations like thyroid carcinoma than those with lesions occurring in nonfixed locations of bone tissue.

## 1 INTRODUCTION

Advances in medical imaging have led to the prevalence of medical image analysis for disease diagnosis, treatment, and prognosis. The main medical imaging modalities include structural imaging (e.g., computed tomography [CT], magnetic resonance imaging [MRI], ultrasound, and optical imaging) that captures anatomic information about an organ or body part and functional imaging (e.g., functional MRI [fMRI] and nuclear medicine [NM]) that reveals both the structural and functional variation in organs and tissues of the human body. As a typical functional imaging technique, bone scintigraphy has been widely used for the diagnosis of bone metastasis caused by a variety of solid tumors mainly including prostate, breast, and lung cancers.^1^ When a primary tumor invades into bone tissue, there will be an area of increased radionuclide uptake.

Single photon emission computed tomography (SPECT) is the most widely used screening procedure for bone scintigraphy in neurology, oncology, and cardiology.^2^ By using radiotracers such as ^99m^Tc‐MDP (methylene diphosphonate), SPECT scintigraphy is capable of providing an assessment of disease stage and severity via visualizing the occupying lesions as areas of increased uptake (i.e., hotspots). Owe to its high disease sensitivity, SPECT scintigraphy has attracted attention from the field of computer‐aided diagnosis/detection. Specifically, the automated models were developed to classify SPECT scintigraphic images using deep learning algorithms.^3^, ^4^, ^5^, ^6^, ^7^, ^8^, ^9^

2D SPECT scintigraphy is characterized by low specificity, mainly caused by the inferior planar spatial resolution, which brings a significant challenge to a manual analysis by physicians for the diagnosis of bone metastasis and other diseases. Moreover, a variety of various nonneoplastic diseases including osteomyelitis, arthropathies, and fractures also present abnormalities on scintigraphic images.^10^ For patients who have undergone recent surgery such as knee or hip replacement, scintigraphy may image false‐positive outcomes.^11^ Therefore, how to accurately classify diseases with SPECT scintigraphic images becomes an urgent problem to be solved in the field of medical image analysis.

Convolutional neural network (CNN) as the mainstream of deep learning techniques has been exploited to develop automated classification models by leveraging their superior capability of automatically extracting features from images at different levels in an optimal way. Existing work mainly focuses on the development of CNN‐based automated classification models for identifying bone lesions metastasized from multiple primary solid tumors,^3^, ^4^ prostate cancer,^5^, ^7^ ^–‐^ ^9^, and breast cancer.^6^ In our previous work, we developed CNN‐based models to identify bone metastasis with thoracic SPECT scintigraphic images^12^ and to segment the metastasized lesions from thoracic SPECT scintigraphic images.^13^

However, the CNN‐based classification of scintigraphic images is still in its infancy. Existing research efforts mentioned above have been made to solve the two‐class classification problem. Precisely, they determine whether an image contains bone metastasis or not. In clinical NM, whole‐body SPECT scintigraphy is often conducted to cover all the bone structures of the human body. As an example, whole‐body SPECT scintigraphy with radiotracers of ^131^ I‐WBS (whole‐body scan) is often used to diagnose clinically thyroid carcinoma. Thus, there may bemultiple kinds of diseases in a given dataset of whole‐body SPECT scintigraphic images. Until now, however, automated multiclass classification of whole‐body SPECT scintigraphic images has not been investigated in the CNN field.

In order to automatically classify diseases with whole‐body SPECT scintigraphy, in this work, we propose a CNN‐based classification network that can automatically identify diseases in whole‐body SPECT scintigraphic images. For doing so, we first augment the dataset of scintigraphic images to solve the problem of limited and imbalanced data that medical image analysis frequently faces. Second, we developed a self‐defined deep CNN by introducing a hybrid attention mechanism to extract hierarchical features from images and classify the high‐level features of concerns (i.e., normal, metastasis, arthritis, and thyroid carcinoma) simultaneously. Finally, a set of clinical whole‐body SPECT scintigraphy images is used to evaluate the performance of the developed classification network by providing comparable analysis between the classical deep networks.

The main contributions of this work can be summarized as follows.

First, we identify the research problem of multidisease classification with the whole‐body SPECT scintigraphy. To the best of our knowledge, this is the first work in the scintigraphic image analysis field.

Second, we convert the problem into an automated multiclass classification of low‐resolution, large‐size images by using a CNN‐based classification network combined with a hybrid attention mechanism.

Finally, we use the clinical SPECT scintigraphic images to evaluate the self‐defined network, with achieving average scores of 0.9801, 0.9795, 0.9791, 0.9933, 0.9792, and 0.9985 for accuracy, precision, recall, specificity, *F*‐1 score, and AUC, respectively.

The rest of this paper is organized as follows. In Section 2, we present the whole‐body SPECT scintigraphic images used in the automated multiclass classification network developed in this work. Experimental evaluations on clinical data of scintigraphic images are reported in Section 3. In Section 4, we conclude this work and point out the future research directions.

## 2 MATERIALS AND METHODS

### 2.1 Dataset

The scintigraphic images used in this work were collected from the Department of NM, Gansu Provincial Hospital from Jan 2017 to Dec 2018, by using a single‐head imaging equipment (GE SPECT Millennium MPR). For patients with suspicious bone metastasis, imaging was performed between 2 and 5 h after intravenous injection of ^99m^Tc‐MDP (20–25 mCi) with a parallel‐beam low‐energy high‐resolution (LEHR) collimator (energy peak = 140 keV, intrinsic energy resolution ≤ 9.5%, energy window = 20%, intrinsic spatial resolution ≤6.9 mm). In postoperative patients with thyroid carcinoma, imaging was performed between 24 and 48 h after oral ^131^I‐WBS (2–5 mCi) with a high energy parallel collimator (energy peak = 364 keV, intrinsic energy resolution ≤ 9.5%, energy window = 10%, intrinsic spatial resolution ≤ 6.9 mm). Two scintigraphic images corresponding to the anterior and posterior views were acquired for each examination. Each scintigraphic image was stored in a DICOM file. The imaging size (apart from the file header and other information) is 256 × 1024 with the pixel size of 2.26 mm, in which each element is represented by a 16‐bit unsigned integer and corrected by using Gaussian filtering (kernel size = 3 and sigma = 1/3 pixel). The acquisition time is 10–15 min for each whole‐body scintigraphic image.

SPECT scintigraphic images were collected from a total of 600 patients aged from 28 to 87 years. Table 1 lists the number of diseases in patients, where one normal class and three diseased classes are included.

**TABLE 1:** Number of diseases in patients involved in the collected SPECT scintigraphic images

	Normal	Bone metastasis	Arthritis	Thyroid carcinoma
Patient	179	117	143	161
Proportion	30%	20%	24%	26%

It is well known that the classification performance of CNN‐based models depends on the size of the dataset, particularly a high classification accuracy always resulting from a large dataset. For this reason, we generate more samples of images by augmenting the original dataset listed in Table 2 with the parameter variation techniques. A concomitant effect of data augmentation is to improve the robustness of the CNN‐based model for alleviating the following problems:

**TABLE 2:** An overview of our SPECT scintigraphic images

	Normal	Bone metastasis	Arthritis	Thyroid carcinoma
Number of images	334	174	252	318
Proportion	31%	17%	23%	29%

First, a change in a patient's position or orientation during the long‐time imaging process that may take up to 5 h is inevitable since, for example, the patient is often startled when the bed shifts to the next scanning position. Automated classification models should be robust enough to deal with displacement and tilt in SPECT scintigraphic images.

Second, the phenomenon of images being not successfully recorded is common in the used dataset. A medical examination has only anterior images, and vice versa, reveals that there are 1078 images from 600 patients. Technical approaches need to be applied to handle the missed SPECT scintigraphic images.

Last, imbalanced samples from different classes may also cause the classifiers to neglect minority class instances and emphasize on majority class, resulting in a skewed classification accuracy.^14^

### 2.2 Data augmentation

A variety of various methods can be used to augment the dataset including the parametric variation and the adversarial learning technique.^15^ Using parametric variation, we can obtain samples that have the same distribution as the original ones with the lower time complexity. In particular, several parametric variation operations such as image mirroring, translation, and rotation are used to augment the dataset described in Table 2. The operations are detailed below.

We first formally represent a DICOM file of a 2D whole‐body SPECT scintigraphic image as a matrix *M_SBS_*:

(1) $$M_{SBS}=\left[\begin{array}{cccc} rd_{11} & \;rd_{12} & \;\ldots & \;rd_{1m}\\ rd_{21} & \;rd_{22} & \;\cdots & \;rd_{2m}\\ \vdots & \;\vdots & \;\ddots & \;\vdots \\ rd_{n1} & \;rd_{n2} & \;\cdots & \;rd_{nm} \end{array}\right],$$

where *rd_ij_* (1 ≤ *i* ≤ *m*, 1 ≤ *j* ≤ *n*) is the radiotracer uptake represented by a 16‐bit unsigned integer, and *m* = 256, *n* = 1024 for 2D whole‐body images acquired by using a GE SPECT Millennium MPR device, with the pixel size of 2.26 mm.

#### 2.2.1 Image mirroring

**FIGURE 1:**
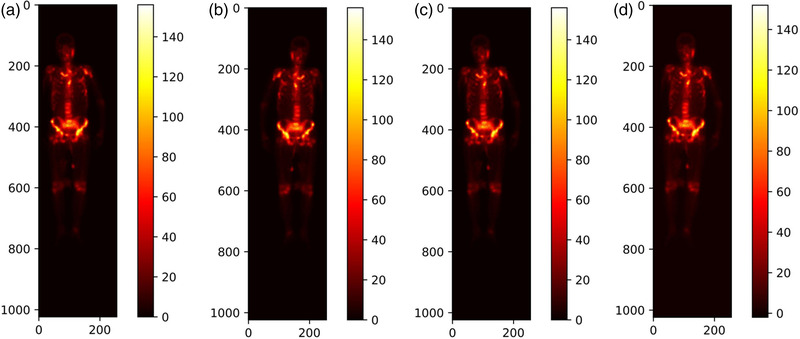
Illustration of mirroring, translating, and rotating whole‐body SPECT scintigraphic image. (a) Original posterior image; (b) mirrored image; (c) translated image; and (d) rotated image by 3^o^ to the right direction

Horizontal mirroring is applied to obtain a posterior counterpart if a SPECT examination has an only anterior view, and vice versa, by reversing this image right‐to‐left along its vertical centerline. Figure 1b depicts the mirrored image of a posterior whole‐body scintigraphic image shown in Figure 1a.

#### 2.2.2 Image translation

For a constant $t\in [0,\;t_{T}]$, an image will be randomly translated by +*t* or ‐*t* pixels in either the horizontal or vertical direction. The parameter *t_T_* is experimentally chosen according to the distribution of the radiotracer uptake of all images in the dataset. Figure 1c shows a resulting example by translating the given image in Figure 1a +5 pixels horizontally.

#### 2.2.3 Image rotation

For a constant $r\in [0,\;r_{T}]$, an image will be randomly rotated by *r* ^o^ in either the left or right direction around its geometric center, where *r_T_* is experimentally determined according to the distribution of the radiotracer uptake of all images in the dataset. Figure 1d shows the obtained image by rotating the image in Figure 1a to the right direction by 3^o^.

These "new" images generated by parametric variation combined with the original ones are grouped into the augmented dataset (see Table 3). The data augmentation alleviates the imbalance of images in different classes as compared to the original ones in Table 2.

**TABLE 3:** An overview of the augmented dataset of SPECT scintigraphic images

	Normal	Bone metastasis	Arthritis	Thyroid carcinoma
Number of images	1660	1582	1500	1788
Proportion	26%	24%	23%	27%

The subsequent section details the process of labeling images to obtain ground truth in the experiments.

### 2.3 Data annotation

In the supervised learning field, labeling image plays a crucial role in training effective and reliable classifiers. However, it is time‐consuming, laborious, and subjective to manually label a 2D whole‐body scintigraphic image due to its poor spatial resolution. The system sensitivity is one of the limitations to label scintigraphic images as a result of its poor spatial resolution and high statistical noise. In this work, we developed an online annotation system based on the open‐source tool LabelMe (http://labelme.csail.mit.edu/Release3.0/) for labeling whole‐body scintigraphic images.

**FIGURE 2:**
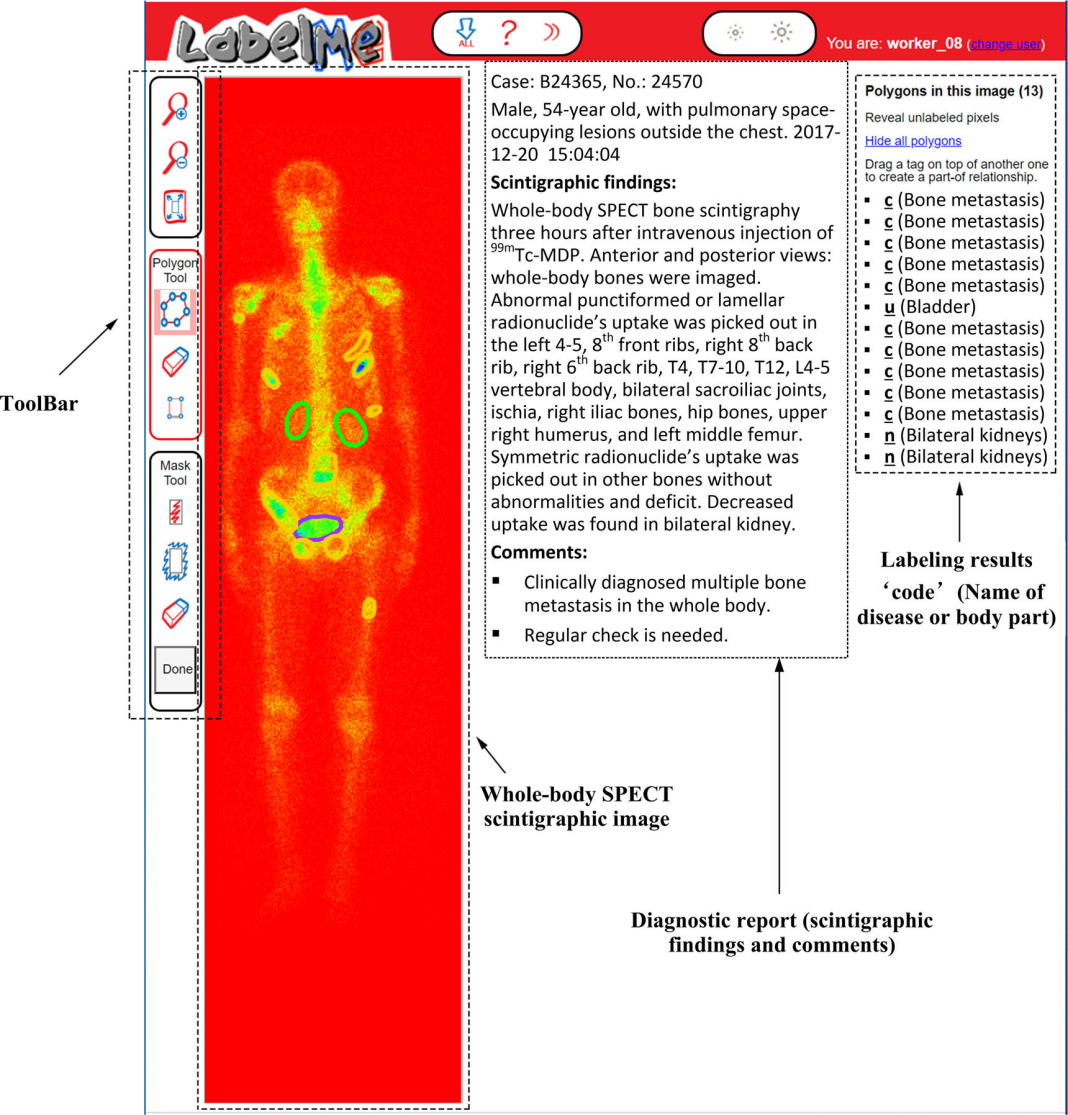
Illustration of labelling a 2D whole‐body SPECT scintigraphic image using the LabelMe‐based annotation system

As illustrated in Figure 2, the DICOM file of a whole‐body SPECT scintigraphic image and the corresponding diagnostic report on scintigraphic findings and comments were imported into the LabelMe‐based annotation system in advance. Three NM physicians from our group manually labeled areas on the image of a DICOM file (RGB format) by using shape tools (e.g., polygons) in the toolbar. The labeled area will be annotated with a code combined with the name of the disease or body part. The results of manual annotation for all images serve as ground truth in the experiments and form an annotation file together, which will be fed into the classification network.

The three NM physicians performed the annotations independently according to the diagnosis reports that were originally issued by the same three NM physicians with the data annotation group and then confirmed by one oncologist. If the majority of physicians (i.e., at least two of them) think that an image is abnormal (i.e., there are one or more lesions of disease in the image), it is labeled as positive (diseased), and negative (normal) otherwise. It is worth noting that each image used in our experiments may contain multiple lesions of the same, rather than different diseases. If there are multiple diseases in a single image, the problem becomes the multiclass (disease), multiobject (lesion) classification of images. It is undeniable that imperfect manual annotation can bring negative impacts on automated classification.

### 2.4 Deep classification network

CNNs are among the successful architectural innovations in deep learning, in which the convolution operator is capable of extracting image features at different abstraction levels. Due to weight sharing, CNNs are now becoming increasingly prevalent in medical image analysis by exploiting the fact that similar structures (e.g., organ, tissue, and lesion) occur in various locations in an image.

In order to extract rich features from low‐resolution, large‐size scintigraphic images, we define an eight‐layer (i.e., eight weight layers) deep classification network as Dscint that detects diseases by classifying images. Table 4 outlines the structure and parameters of the Dscint network.

**TABLE 4:** Structure and parameters of the self‐defined deep classification network Dscint

Layer	Configuration
Convolution	11 × 11, 16, S = 4, P = 2
Pooling	MaxPool(3), S = 2
	Attention module
Convolution	5 × 5, 16, S = 1, P = 2
	BatchNorm
Pooling	MaxPool(3), S = 2
Convolution	3 × 3, 24, S = 1, P = 1
Convolution	3 × 3, 24, S = 1, P = 1
Convolution	3 × 3, 24, S = 1, P = 1
	BatchNorm
Pooling	MaxPool(3), S = 1
Fully connected	1024
Fully connected	1024
Softmax	4

Abbreviations: MaxPool, max pooling; P, padding; S, stride.

#### 2.4.1 Weight layer

There are five convolutional layers and three pooling layers in the self‐defined network. The convolution operation denoted as < kernel_size = *n* × *n*, channel_number, stride_size, padding_size > produces feature maps. An original input of a 256 × 1024 whole‐body image is convolved with each 11 × 11 filter in the first convolutional layer to calculate a feature map made of neurons. The subsequent convolutional layers take the feature maps of immediately previous layers as inputs to convolve with each filter. Pooling operation is used to down‐sample the feature maps from the convolutional layer before it. The max‐pooling used in Dscint partitions a feature map into a set of sub‐regions with the size of 3 × 3, and outputs the maximum value for each of such sub‐regions.

#### 2.4.2 Hybrid attention module

**FIGURE 3:**
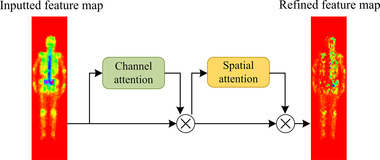
Hybrid attention module with the channel and spatial attention in the self‐defined Dscint network

After the first pooling layer, we introduced the hybrid attention module to improve Dscint in a way that focuses on more important regions (i.e., lesions) on the 2D feature maps by considering the important information. The cascaded hybrid attention module (see Figure 3) using the channel and spatial attention mechanisms can compute complementary attention by focusing on “what” (channel attention) and “where” (spatial attention), respectively.^16^

Let *F* denote the input of a 2D feature map to the channel attention sub‐module. We can achieve a 1D output ₣, which will be further processed by the spatial attention sub‐module to output a refined 2D feature map *M* according to Equation (2).

(2) $$M=f_{S}\left(f_{C}\left(F\right)⊗F\right)⊗F,$$

where ⊗ is the element‐wise multiplication, and *f_C_* and *f_S_* denote the channel and spatial function, respectively, which will be defined in Equations (3) and (4).

(3) $$\begin{array}{c} f_{C}\left(F\right)=\sigma \left(\mathrm{MLP}\left(\mathrm{AvgPool}(F)\right)+\mathrm{MLP}\left(\mathrm{MaxPool}(F)\right)\right), \end{array}$$

(4) $$f_{S}\left(F\right)=\sigma \left(f^{k\times k}\left(\left[\mathrm{AvgPool}(F);\mathrm{MaxPool}(F)\right]\right)\right),$$

where *σ* is the sigmoid function, MLP is the multilayer perceptron, AvgPool (MaxPool) is the average (max) pooling, and *f ^k^* ^×^ *^k^* is a convolutional operation with the kernel size of *k* × *k*.

#### 2.4.3 BatchNorm layer

Batch normalization^17^ is utilized in the layers after the second and fifth convolutional layers in Dscint. It aims to accelerate network training by making normalization a part of the model architecture and performing the normalization for each training mini‐batch. With batch normalization, we can therefore use much higher learning rates and be less careful about initialization.

#### 2.4.4 Fully connected layer

We use two fully connected layers to make a non‐linear combination of the selected features at the end of the network. Within each fully connected layer, neurons are fully connected to all activations in the previous layer, to produce an output in the form of a simple vector. The activations are often calculated with matrix multiplication, followed by a bias offset.

#### 2.4.5 Softmax layer

We use the Softmax function in the network output layer with a real number, four unordered categories (i.e., normal, metastasis, arthritis, and thyroid carcinoma). Let *x_j_* be the input to *j*th output node. The Softmax function *f*(*x_j_*) calculates a score of this output node according to Equation (5).

(5) $$f\left(x_{j}\right)=\frac{e^{x_{j}}}{\sum_{i=1}^{n}e^{x_{i}}},$$

where *n* is the number of output nodes. We have 0 ≤ *f*(*x_j_*) ≤ 1 and ∑ *f*(*x_j_*) = 1.

## 3 RESULTS

This section reports the experimental results of the self‐defined classification network of Dscint on clinical whole‐body SPECT scintigraphic images as provided in Tables 2 and 3.

### 3.1 Experimental setup

The evaluation metrics used in this work include accuracy, precision, recall, specificity, *F*‐1 score, and AUC (area under ROC curve). They are, respectively, defined in Equations ((6), (7), (8), (9), (10)).

(6) $$\mathrm{Accuracy}=\frac{\mathrm{TP}+\mathrm{TN}}{\mathrm{TP}+\mathrm{TN}+\mathrm{FP}+\mathrm{FN}},$$

(7) $$\mathrm{Precision}=\frac{\mathrm{TP}}{\mathrm{TP}+\mathrm{FP}},$$

(8) $$\mathrm{Recall}=\frac{\mathrm{TP}}{\mathrm{TP}+\mathrm{FN}},$$

(9) $$\mathrm{Specificity}=\frac{\mathrm{TN}}{\mathrm{TN}+\mathrm{FP}},$$

(10) $$F-1\;\mathrm{score}=2\times \frac{\mathrm{Precision}\times \mathrm{Recall}}{\mathrm{Precision}+\mathrm{Recall}},$$

where the notations are TP = true positive, TN = true negative, FP = false positive, and FN = false negative.

A classifier should show both a high true positive rate (TPR = Recall) and a low false‐positive rate (FPR) simultaneously. The ROC curve shows the true positive rate (*y*‐axis) against the false positive rate (*x*‐axis), and the AUC value is the area under the ROC curve. As a statistical explanation, the AUC value is equal to the probability that a randomly chosen positive image is ranked higher than a randomly chosen negative one. Thus, the closer to 1 the AUC value is, the higher performance the classifier achieves.

Each dataset (i.e., the original one in Table 2 and its augmented one in Table 3) was randomly divided into two parts, i.e., training subset and the test subset. Images including the augmented ones from the same patient were not divided into the different subsets because they would show similarities. The ratio of the training subset and the test subset is about 7: 3. Images samples in the training subsets are for training the classification network while the samples in the test subsets are used to test the performance of the network. The trained classifier was run 10 times on the test subset in order to reduce the effects of randomness. For each of the defined metrics above, the final output of the classifier is the average of the 10 running results. The experimental results reported in the next section are the average ones unless otherwise specified.

The parameter settings of Dscint are provided in Table 5. The experiments were run in Tensorflow 2.0 on an Intel Core i7‐9700 CPU with 32GB RAM running Windows 10.

**TABLE 5:** Parameter settings of the self‐defined classification network of Dscint

Parameter	Value
Learning rate	10^–3^
Weight decay	10^–4^
Batch size	4
Epoch	300
Iteration	1200

### 3.2 Experimental results

**FIGURE 4:**
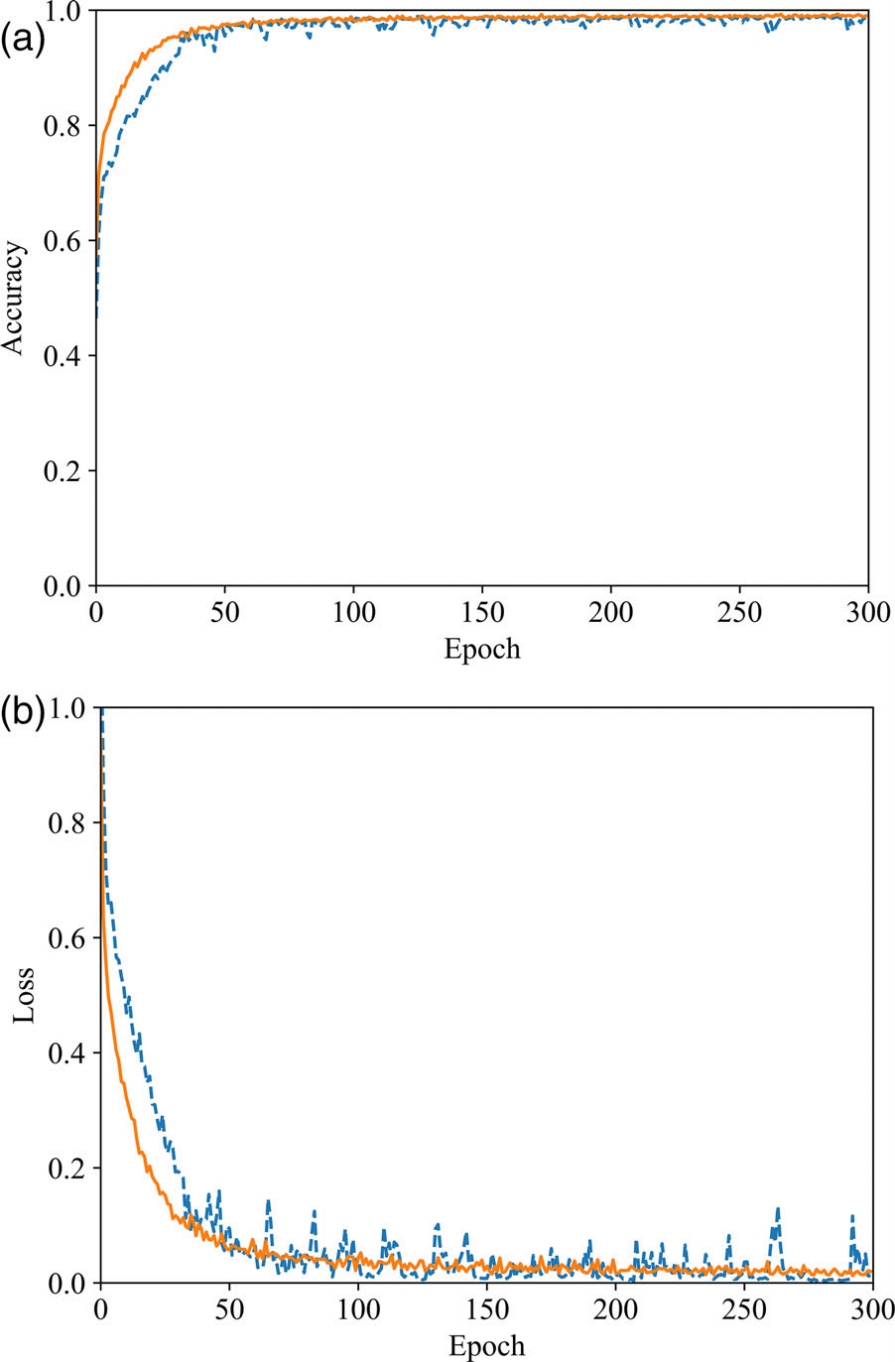
Illustration of training Dscint on the original (blue) and augmented (orange) datasets. (a) Accuracy curves and (b) loss curves

Figure 4 depicts the accuracy and loss curves obtained by training Dscint on the original (blue) and augmented (orange) datasets, respectively. The training time is 2.87 (14.62) h for the original (augmented) dataset. We can see that the data augmentation contributes largely to improving both accuracy and stability in a way that significantly higher performance than on the original dataset during the first 50 epochs are obtained.

To examine the classification performance of Dscint on the test datasets, Table 6 reports the scores of the evaluation metrics as defined in Equations ((6), (7), (8), (9), (10)). The performance is consistent with the ones in the training stage, with higher values of metrics being obtained on the augmented dataset than the original one. This reveals that Dscint performed well in classifying whole‐body SPECT scintigraphic images.

**TABLE 6:** Scores of evaluation metrics obtained by Dscint on the test samples in both original and augmented dataset

	Accuracy	Precision	Recall	Specificity	*F*‐1 score
Original data	0.8519	0.8599	0.8257	0.9489	0.8362
Augmented data	0.9801	0.9795	0.9791	0.9933	0.9792

**FIGURE 5:**
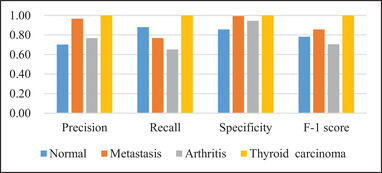
Quantitative performance obtained by Dscint on test samples in the augmented dataset with average scores of evaluation metrics for different classes of concerns

With the test samples in the augmented dataset in Table 3, we further analyze the classification performance of Dscint on distinguishing various diseases by reporting scores of evaluation metrics for classes of concerns in Figure 5.

From the classification performance as shown in Figure 5, we can see that Dscint performs the best for thyroid carcinoma but the worst for arthritis (*F*‐1 = 0.7059). It is suitable to distinguish between classes especially the diseased classes (specificity ≥ 0.94 for metastasis, arthritis, and thyroid carcinoma). However, misclassification occurs not only between the diseased classes but also between the normal and diseased classes. The reasons for this are as follows.

First, the fixed location of thyroid carcinoma improves the deep classification network of Dscint. Focusing on the region of interest (i.e., thyroid) in scintigraphic images, Dscint extracts rich hierarchical features from these regions, producing high classification performance. On the contrary, arthritis can occur at any site of the skeleton. As such, it makes Dscint difficult to extract rich features of arthritic lesions from the small‐scale dataset (the augmented dataset is still a small‐scale dataset).

Second, whole‐body SPECT scintigraphy is characterized by the inferior planar spatial resolution and large variation of radiotracer uptake from patient to patient. The low quality of images makes it very challenging to distinguish the disease‐caused increase in tracer uptake and the normal variation of uptake. Moreover, the normal variation in uptake relates to the bony metabolic activity negatively correlated with age.^18^

**FIGURE 6:**
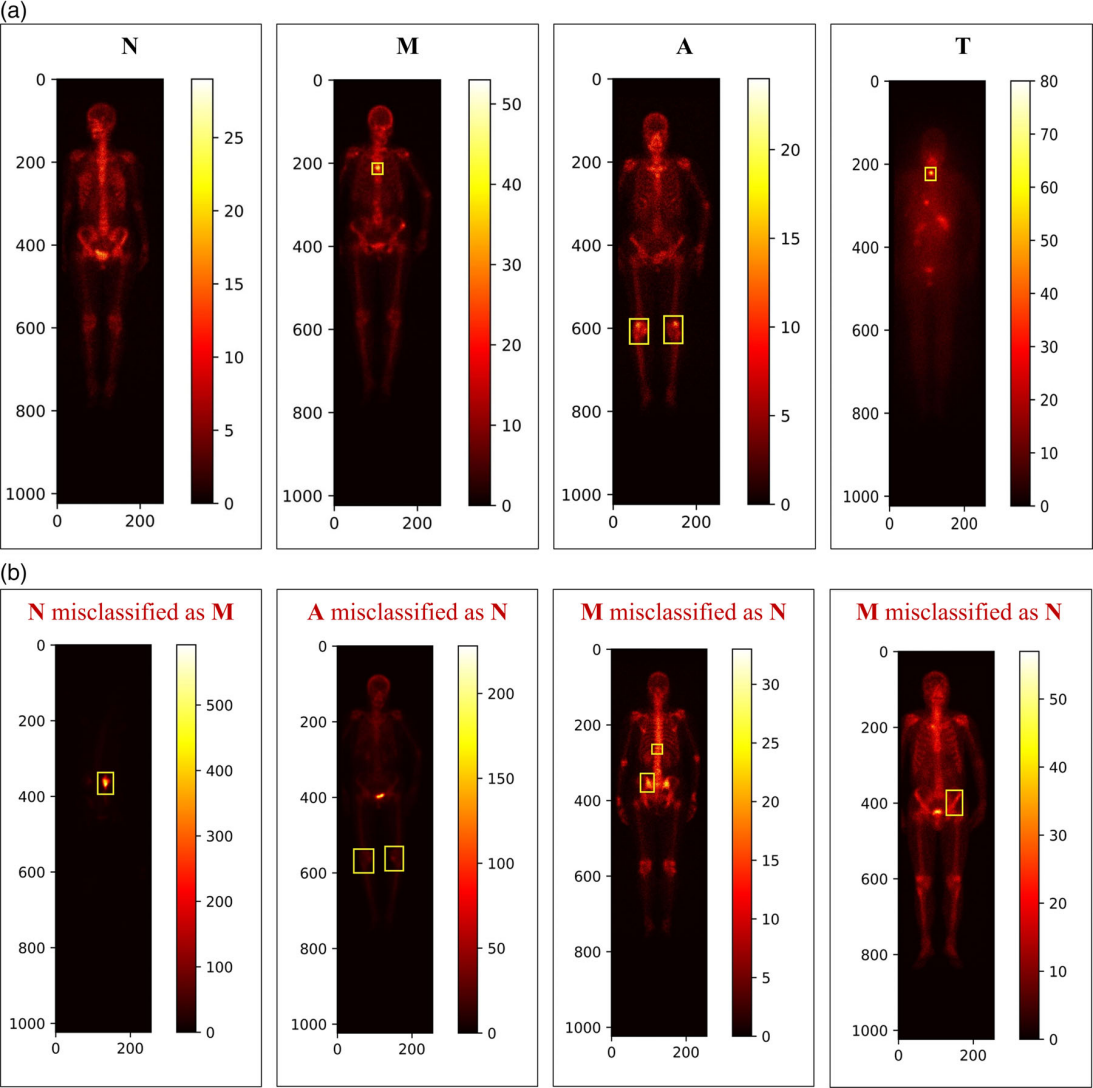
Examples of misclassified whole‐body SPECT scintigraphic images with N = Normal; M = Metastasis; A = Arthritis; and T = Thyroid carcinoma. (a) Correctly classified images and (b) wrongly classified images

We illustrate examples of classified whole‐body SPECT scintigraphic images with the correctly classified ones in Figure 6a and the wrongly classified ones in Figure 6b. The possible reasons for such a misclassification were provided by one experienced NM physician from our group who completed data annotation, which is detailed below. The possible solutions are also provided.

#### 3.2.1 Patient‐related factors

The common patient‐related artifacts mainly include the extravasation of radiopharmaceuticals at the site of injection, urinary contamination, and soft tissue uptake.^10^ These factors may occasionally cause confusion with an abnormality in bone tissue. They bring a large challenge to the automated classification of scintigraphic images with CNNs‐based models. This is the reason why the normal image was misclassified as metastasis in Figure 6b, denoting as “*N* misclassified as *M*.” Image cropping to extract areas of interest would be needed before performing a classification task.

#### 3.2.2 Low contrast

For patients with mild arthritis, for example, the lower tracer uptake in the lesions may easily be misclassified as normality, which is denoted as “*A* misclassified as *N*” in Figure 6b. The methods such as normalization should be applied to deal with the low contrast problem of whole‐body SPECT scintigraphic images.

#### 3.2.3 Postprocessing

The large variation of radiotracer uptake between patients requests that personalized features should be extracted from a large‐size dataset of scintigraphic images. As mentioned previously, the normal variation of uptake relates to the bony metabolic activity that is correlated negatively with age.^18^ The metastasized images acquired from two patients aged 83 and 76 years, respectively, were misclassified as normal in Figure 6b, denoting as “*M* misclassified as *N*”. A postprocessing stage is needed to be integrated into the automated classification network to examine the asymmetric uptake. This is because the irregular, asymmetric or eccentric radiotracer uptake in scintigraphic images may be towards malignant involvement.^11^

### 3.3 Classification performance comparison

The comparative analysis of classification performance was performed between Dscint and several classical CNNs including AlexNet,^19^ ResNet,^20^ VGG‐16,^21^ Inception‐v4,^22^ and DenseNet.^23^ An overview of comparing these classical networks is given in Table 7, in terms of the number of their weight layers, filter shape, activation function, and optimizer. The parameter settings of these models are the same as Dscint (see Table 5).

**TABLE 7:** An overview of the classical CNNs‐based models used for comparative analysis

	Weight layer	Filter	Activation	Optimizer
AlexNet	8	11 × 11, 5 × 5, 3 × 3	ReLU	Adam
ResNet	18	3 × 3	ReLU	Adam
VGG‐16	16	3 × 3	ReLU	Adam
Inception‐v4	14 Inception	3 × 3, 1 × 1, 1 × 7, 7 × 1, 1 × 3, 3 × 1	ReLU	Adam
DenseNet	121	1 × 1, 3 × 3	ReLU	Adam

The scores of evaluation metrics obtained by all networks are reported in Table 8. From which we can see that Dscint outperforms all the classical CNNs. Specifically, the deepest network Inception‐v4 obtains the worst classification performance. We can conclude that the network depth is related inversely to the classification performance, which is mainly due to the limited data of scintigraphic images.

**TABLE 8:** Evaluation metrics obtained by six models on test samples

	AlexNet	ResNet	VGG‐16	Inception‐v4	DenseNet	Dscint
Accuracy	0.9652	0.9314	0.9550	0.8384	0.9371	0.9801
Precision	0.9641	0.9316	0.9538	0.8367	0.9391	0.9795
Recall	0.9643	0.9293	0.9556	0.8348	0.9332	0.9791
Specificity	0.9884	0.9767	0.9850	0.9420	0.9781	0.9933
F‐1 score	0.9715	0.9303	0.9541	0.8345	0.9309	0.9792

**FIGURE 7:**
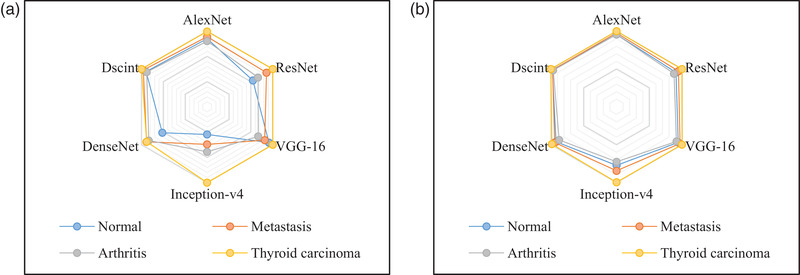
A comparison of evaluation metrics obtained by CNNs‐based classification models on test samples in the augmented dataset. (a) Specificity and (b) *F*‐1 score

The radar maps in Figure 7 demonstrate that CNN‐based models achieve the best classification performance (Specificity and *F*‐1 score) for thyroid carcinoma with the largest hexagon in yellow. The outermost points on hexagons denoting Dscint reveal that Dscint is the best‐performing model for automated classification of scintigraphic images on the augmented dataset. We can also see that the arthritic images challenge all classification networks and the normal images are easy to be misclassified into other classes. However, the decrease in overall performance was caused by the arthritic image.

**FIGURE 8:**
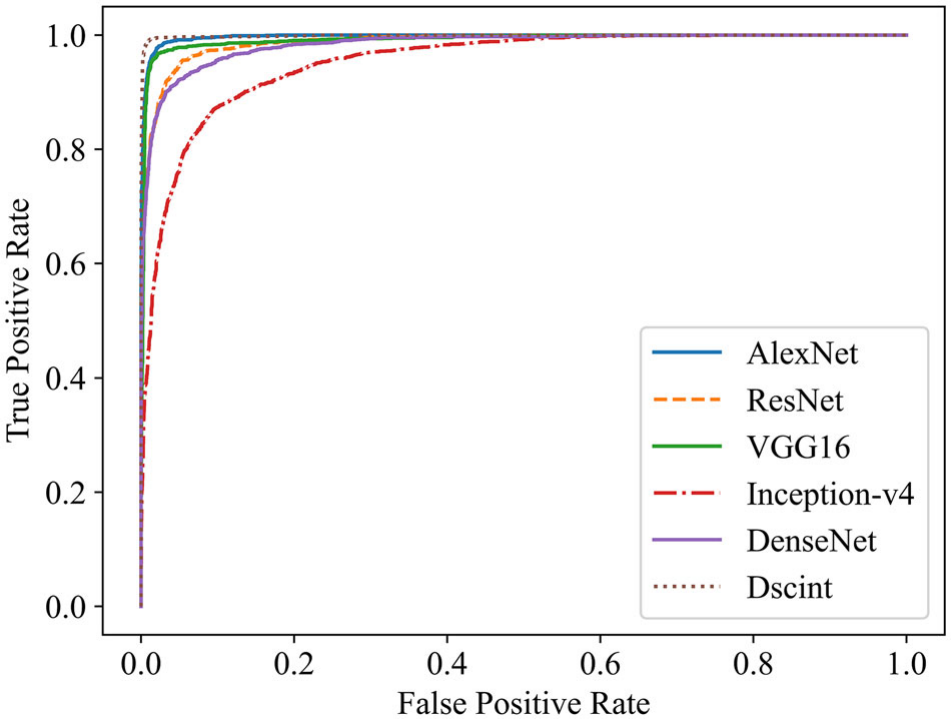
ROC curves obtained by six models on test samples of the augmented dataset in Table 3

The ROC curves and the corresponding AUC values obtained by six models are shown in Figure 8 and Table 9, respectively.

**TABLE 9:** AUC values obtained by six models on test samples of the augmented dataset in Table 3

	AlexNet	ResNet	VGG‐16	Inception‐v4	DenseNet	Dscint
AUC	0.9973	0.9877	0.9921	0.9532	0.9838	0.9985

**FIGURE 9:**
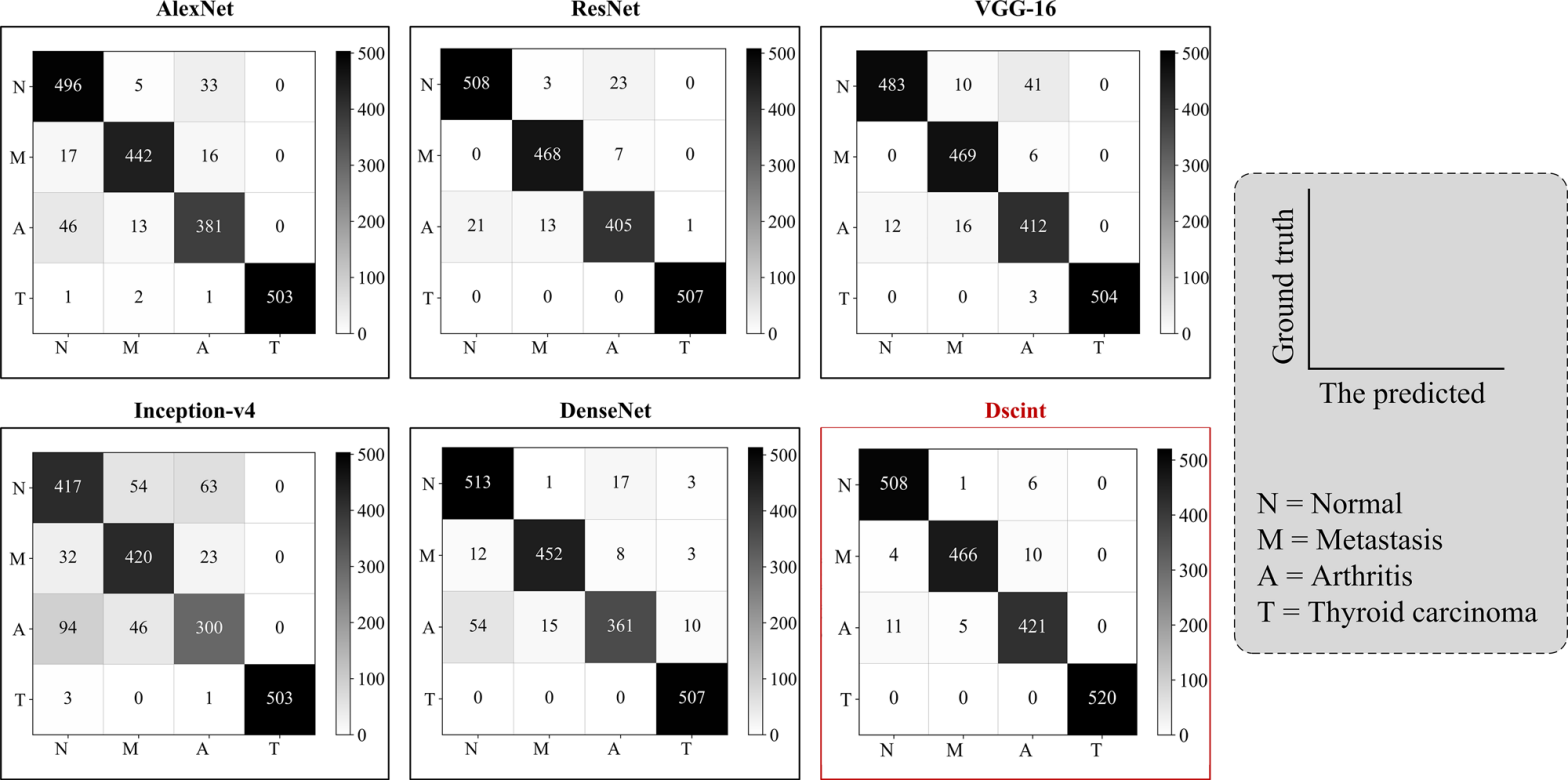
Confusion matrices obtained by six models on test samples in the augmented dataset

Similarly, we present in Figure 9 the confusion matrices obtained by these models on classifying images of concerns with test samples in the augmented dataset.

It can be found that classical classification models share the same source of misclassification as Dscint, i.e., the difficulty of distinguishing between the normal/metastasized and arthritis classes. Eleven arthritic images were incorrectly classified as normal while ten metastasized images were misclassified as arthritis images by Dscint.

In a word, our self‐defined classification network of Dscint can detect various diseases in whole‐body scintigraphic images by automatically classifying these images of concern. Specifically, the Dscint network performs well on the task of automated classification with images that contain lesions occurring in relatively fixed areas of images. By contrast, all deep networks achieve lower performance in terms of the defined evaluation metrics for classifying those diseases that can occur in any location (e.g., arthritis and multiple bone metastasis) with the small‐scale dataset.

## 4 CONCLUSIONS

Targeting the automated classification of diseases with SPECT scintigraphy, we have developed a CNN with the hybrid attention mechanism in this work. Parametric variation was first conducted to augment the dataset of original images. A deep classification network called Dscint has been developed to automatically extract features from images and classify these features into classes. Clinical whole‐body scintigraphic images were utilized to evaluate the developed network. Experimental results have demonstrated that our self‐defined network performs well in detecting diseases. The analysis has also been conducted for comparing Dscint with several classical models. The results reveal that our method can be used for automated detection of diseases including arthritis, metastasis, and thyroid carcinoma.

In the future, we plan to extend our work in the following directions.

First, we intend to collect more data of SPECT scintigraphic images, laboratory findings, and textual data to improve the proposed classification network. Hopefully, a robust, effective, and efficient computer‐aided diagnosis system will be developed.

Second, we attempt to develop deep learning‐based methods that can classify whole‐body SPECT scintigraphic images with multiple lesions from various diseases that may present in a single image.

Last, we plan to design different network structures by using network architecture search^24^ and deep supervision learning^25^ techniques to accurately diagnose diseases with multisource medical data.

## CONFLICT OF INTEREST

The authors declare no conflict of interest.

## INFORMED CONSENT STATEMENT

The study was conducted according to the guidelines of the Declaration of Helsinki, and approved by the Ethics Committee of Gansu Provincial Hospital (Lot No.: 2020–199, August 26, 2020). Patient consent was waived due to the anonymous nature of the data.

## Data Availability

Due to the ethical and legal restrictions on the potential health information of patients, the data are not available openly. Dataset can only be accessed upon request by emailing Ms. Rong Wang (1160023677@qq.com) who is on behalf of the Ethics Committee of Gansu Provincial Hospital.
